# A new singular species of *Croscherichia* Pardo Alcaide, 1950 (Coleoptera, Meloidae, Mylabrini) from arid zones of eastern Morocco

**DOI:** 10.3897/zookeys.885.34308

**Published:** 2019-11-04

**Authors:** José L. Ruiz, Alexandre François, Mario García-París

**Affiliations:** 1 Instituto de Estudios Ceutíes. Paseo del Revellín, 30. 51001 Ceuta. Spain Instituto de Estudios Ceutíes Ceuta Spain; 2 Emirates Center for Wildlife Propagation (ECWP). B.P. 47. MA 32250 Missour. Morocco Emirates Center for Wildlife Propagation Missour Morocco; 3 Museo Nacional de Ciencias Naturales (MNCN-CSIC). c/ José Gutiérrez Abascal, 2. 28006 Madrid. Spain Museo Nacional de Ciencias Naturales Madrid Spain

**Keywords:** *
Ammabris
*, arid steppes, *
Atriplex
*, biodiversity, blister beetle, Hammada, morphology, North Africa, taxonomy

## Abstract

A new species of blister beetle (Coleoptera, Meloidae, Mylabrini), *Croscherichia armass* Ruiz, François & García-París, **sp. nov.**, is described from the arid steppes of eastern Morocco (Missour, Boulemane Province). The new species presents traits shared with both *Croscherichia* and desert species of the genus *Ammabris*, making it morphologically singular. Conspicuous external similarities (coloration pattern, shape of the mandibles, setation) between *C. armass***sp. nov.** and *Ammabris* allow the two to be easily confused. However, *C. armass***sp. nov.** can be readily distinguished from all other *Croscherichia* species by the following traits: reddish-orange legs with dark tarsi; relatively short black antennae with the proximal-most three to four antennomeres of each antenna having a reddish-brown coloration; dense and silvery body setation that lies over most of the body integument; straight and pointed outer mandible margins that protrude from the labrum; a mesosternum with an angulate anterior margin; a short, subcylindrical, and weakly spatulate external metatibial spur that is truncated obliquely at the apex. *Croscherichia armass***sp. nov.** is only known from three localities in the arid Hammada steppes, which are located within the Quaternary alluvial plains of the Muluya river valley. Live specimens of *C. armass***sp. nov.** were found in flight and actively feeding on *Atriplex halimus* (Chenopodiaceae) flowers at the end of summer (mid-September). The phenology of *C. armass***sp. nov.** is exceptional as no other Mylabrini species known from eastern areas of Morocco are active in late summer.

## Introduction

Mylabrini Laporte 1840, which is distributed over most of the Old World (Palaearctic, Oriental and Afrotropical regions), is the most diversified tribe within the family Meloidae. Mylabrini is comprised of about 760 species distributed in 12 genera (Bologna 1991, Bologna and Pinto 2002, Pan et al. 2013, Pan and Bologna 2014, Salvi et al. 2018). Morphological and molecular analyses support the monophyly of Mylabrini (Bologna 1991, Bologna and Pinto 2001, Bologna et al. 2005, 2008a, 2008b, Pan et al. 2013, Salvi et al. 2018). However, taxonomic assignments made within the tribe have been problematic, with many issues still unresolved. Most of the species within Mylabrini (82%) are assigned to one of two genera: *Hycleus* Latreille, 1817 or *Mylabris* Fabricius, 1775, with around 450 and 175 species, respectively (e.g., Bologna 1991, 2000, Bologna and Pinto 2002, Bologna et al. 2011, Pan and Bologna 2014). Although the systematics of *Hycleus*, which is likely polyphyletic, have not been adequately addressed, the monophyly of *Mylabris* was recently confirmed using molecular data, and its phylogeny resolved to the subgenus level (Salvi et al. 2018).

In contrast to *Hycleus* and *Mylabris*, *Croscherichia* Pardo Alcaide, 1950 is a medium-sized genus currently comprised of 18 described species, whose internal phylogeny based on morphological traits and a largely undisputed taxonomy is well recognized (Biondi and Bologna 1991, Bologna and Coco 1991). *Croscherichia* is widely distributed in northern Africa, the northern Sahel region, the Middle East, the Arabian Peninsula, and western India (Bologna 1991, Bologna and Coco 1991, Bologna and Pinto 2002, Bologna 2008). The presence of the genus in western Europe (particularly, the Iberian Peninsula and the Balearic Islands), based on old records of *Croscherichia paykulli* (Billberg, 1813), has been rejected by several authors (García-París and Ruiz 2005, García-París et al. 2010), as has its presence in Turkestan (Bologna and Coco 1991, Bologna and Pinto 2002, Bologna 2008).

In its current sense, *Croscherichia* [type species: *Mylabris circumflexa* Chevrolat, 1840 (= *Mylabris paykulli* Billberg, 1813), by monotypy] is monophyletic. This genus, with its sister group *Mimesthes* Marseul, 1872, is characterized by the synapomorphic condition of its external metatibial spur, which is spatulate in both groups (Bologna et al. 2005, Salvi et al. 2018). Some ecological and developmental studies of *Croscherichia* have been reported, including the description of first-instar larvae of a few species (Cros 1917, 1927, 1940, MacSwain 1956, Bologna and Coco 1991). First-instar *Croscherichia* larvae present a few traits typical of phoretic taxa, which are exceptional for Mylabrini; however, their biology is still largely unknown (Bologna and Pinto 2001, Bologna et al. 2005).

Bologna and Coco (1991) established the generic limits of *Croscherichia* by reassigning to it some species previously included in *Hycleus* (= *Gorrizia* Pardo Alcaide, 1954) or in *Mylabris*. The species of *Croscherichia* are characterized by the following morphological diagnostic characters: a medium to large size (8–22 mm); black or reddish-brown integument, without a metallic shine; large convex eyes; antennae with 11 elongate antennomeres (rarely, X and XI almost fused) that are not flabellate or compressed and that do not have a marked distal thickening; mesopleurae that are not bordered along the anterior margins; an angled mesosternal suture; mesosternum without a modified middle-anterior area (*scutum*) or divergent groves; an elytral design consisting of sinuated transverse bands or rounded spots; a cylindrical external metatibial spine that is truncated obliquely at the apex and usually broadened and spatulate (spoon-like) distally; aedeagus with elongate, narrow parameres (=gonoforceps), with straight and equally elongated distal lobes; and a median lobe with two subequal small teeth (harpagae), with the distal tooth far from apex (Pardo Alcaide 1950, 1954a, Bologna and Coco 1991, Salvi et al. 2018). Of the species currently included in *Croscherichia*, the assignment of one is particularly controversial: a morphologically peculiar species from the Arabian Peninsula (Bologna and Pinto 2002) that was originally described as Mylabris (Gorrizia) sonyae Kaszab, 1983 but that shares a combination of traits with *Hycleus* and *Croscherichia* (Kaszab 1983, Bologna and Pinto 2002, Bologna and Turco 2007, Batelka and Geishardt 2009). Therefore, its inclusion in *Croscherichia* needs to be re-evaluated (Bologna and Turco 2007).

**Figure 1.**
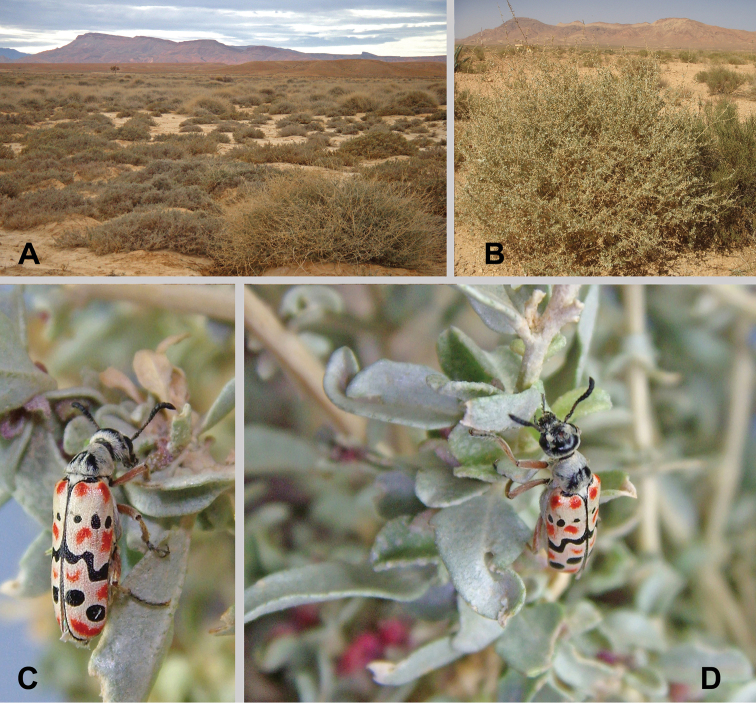
Habitat and live specimens of *Croscherichia armass* sp. nov. **A** thickets of saltbush (*Atriplex halimus*) in an alluvial silt plain of the Al Baten area between Missour and Outat el Haj **B** detail of *Atriplex halimus* L. where the species is usually found. Emirates Center for Wildlife Propagation (ECWP, Missour) **C, D** active specimen on *Atriplex halimus* L. (ECWP, Missour). Photographs by AF.

**Figure 2.**
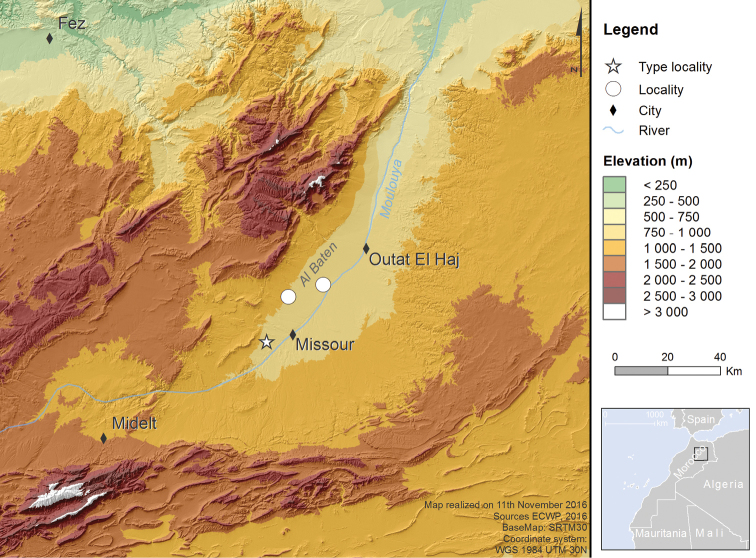
Map of the Missour area in eastern Morocco, showing the type locality of *Croscherichia armass* sp. nov. (star) and the two other localities where the species occurs (circles).

In this work, a new species of *Croscherichia* is described from arid zones of central-eastern Morocco (Missour region) (Figs 1A, B; 2). The new species presents morphological traits that are different from those of any other *Croscherichia* species, and similar to *C. sonyae* (Kaszab, 1983), singular traits that resemble those in other Mylabrini genera. The shape of the mandibles, the macrosculpture and setation of the pronotum, and the elytral design of the new species (Fig. 1C, D) closely resemble those of some North African species of the genus *Ammabris* Kuzin, 1954 [*Ammabris* was considered a subgenus of *Mylabris* but is now treated as a genus following the proposal by Salvi et al. (2018)].

The fauna of Mylabrini is relatively well known in Morocco (e.g., Martínez de la Escalera 1909, 1914, Pardo Alcaide 1954a, 1954b, 1954c, 1961, 1969, Kocher 1956, 1964, 1969, Ruiz 2000, 2004, Ruiz and García-París 2008, 2014, Bologna 2008); therefore, it is remarkable that the new species remained undescribed for so long, an indication of the ongoing lack of zoological knowledge for the arid and semi-arid eastern regions of the country. The imaginal phenology of the new species is restricted to the end of summer, an unusual period for entomological surveys of North African arid zones, which may explain its late discovery.

## Material and methods

A total of 19 dry-preserved (5 males and 14 females) and 4 ethanol-preserved (2 males and 2 females) specimens were used to describe the new species of *Croscherichia*. The specimens were collected from three nearby localities (separated by a maximum distance of 35 km) in the region of Missour (Fès-Boulemane, Morocco) (Fig. 2). Of the 23 studied specimens, 7 were collected in pitfall traps (in 2002) and 16 by hand while they were actively feeding on *Atriplex halimus* L. (in 2015). Samplings were carried out as part of several projects on the entomological diversity of arid areas in eastern Morocco that were sponsored by the Emirates Center for Wildlife Propagation (ECWP). The type series has been deposited in the following collections: the ECWP collection (Missour, Morocco) (9 paratypes, all dry-preserved); the Museo Nacional de Ciencias Naturales (MNCN-CSIC) (Madrid, Spain) (the holotype and 9 paratypes, 5 dry-preserved and 4 in ethanol); and the J. L. Ruiz collection (JLR) (Ceuta, Spain) (4 paratypes, all dry-preserved).

Interspecific comparisons were performed using the material indicated below (308 specimens), including specimens of all known species of *Croscherichia* except *C. femorata* (Klug, 1845) from Arabia, *C. quadrizonata* (Fairmaire, 1875) from eastern Algeria, Tunisia, and Libya, and the aforementioned problematic *C. sonyae*. The diagnostic morphological traits of these three species were extracted from Kaszab (1983), Bologna and Coco (1991), and Bologna and Turco (2007). Additional acronyms or abbreviations: HNHM, Hungarian Natural History Museum (Budapest, Hungary) and ex./x., exemplar/s.

Material examined: ***Croscherichia albilaena*** (Bedel, 1899): ALGERIA: Biskra, L. Bleuse, Mai 1885 (Slg. R. Oberthür, Coll. E. Martin, Eing. Nr. 4 1956, *Croscherichia albilaena* Bedel, Dr Kaszab det. 1957): 1 ex. (HNHM). ***Croscherichia bedeli*** (Bleuse, 1899): ALGERIA: Aïn-Sefra, Mai 1896, A. Chobaut (Coll. Reitter, *Mylabris bedeli* Bleuse, det. Dr Kaszab, *Croscherichia bedeli* Bleuse, Dr Kaszab det. 1957): 4 exx. (HNHM). MOROCCO: Figuig, 29-V-1991, G. Chavanon leg.: 2 exx. (JLR); Figuig, 21-V-1993, G. Chavanon leg.: 3 exx. (JLR); Figuig, erg à *Aristida pungens*, 10-V-1997, G. Chavanon leg.: 15 exx. (JLR); Figuig, 14-V-1999, G. Chavanon leg.: 9 exx. (JLR). ***Croscherichia delarouzei*** (Reiche, 1865): SYRIA: Syrien, Kafa, Reitter (Coll. Reitter, *Croscherichia delarouzei* Reich., Dr Kaszab det. 1957): 1 ex. (HNHM). ***Croscherichia fulgurita*** (Reiche, 1866): ALGERIA: Aïn-Sefra (Oran), L. Bleuse (Coll. Reitter, *Mylabris fulgurita* Reiche, det. Dr Kaszab, *Croscherichia fulgurita* Reiche, Dr Kaszab det. 1957): 1 ex. (HNHM). MOROCCO: Figuig, 14-V-1999, G. Chavanon leg.: 1 ex. (JLR). ***Croscherichia gilvipes*** (Chevrolat, 1840): EGYPT: Le Caire Hénon (Slg. R. Oberthür, Coll. E. Martin, Eing. Nr. 4 1956, *Croscherichia angulata* Klug, Dr Kaszab det. 1959): 1 ex. (HNHM). MOROCCO: Bouârfa, 20-V-1993, G. Chavanon leg.: 3 exx. (JLR); Bouârfa, 23-V-1993, G. Chavanon leg.: 5 exx. (JLR); Figuig, erg à *Aristida pungens*, 10-V-1997, G. Chavanon leg.: 1 ex. (JLR); Route Bouàrfa-Figuig, 32.47915/-1.74761, 1170 m, 24-V-2015, A. François, M. García-París & J.L. Ruiz leg.: 2 exx. (ECWP & JLR). TUNISIA: Tunisia Reitter (Coll. Reitter, *Croscherichia gilvipes* ab. Dr Kaszab det. 1957): 1 ex. (HNHM). ***Croscherichia goryi*** (Marseul, 1870): IRAN: Midjan, 9-V-1969, Paz-Hasch: 1 ex. (HNHM). PALESTINE: Palestine, Wadi Fukra, IV-1945, leg. Bylinski-Salz (*Mylabris goryi* Mars., det. Kaszab 1956, *Croscherichia goryi* Mars., Dr Kaszab det. 1957): 1 ex. (HNHM). ***Croscherichia litigiosa*** (Chevrolat, 1840): MOROCCO: Bouârfa, 32.53565/-2.55283, 8-V-2008, S. Touil leg.: 7 exx. (ECWP); Bouârfa, 32.40921/-2.59711, 15-V-2008, S. Touil leg.: 8 exx. (ECWP); Bouârfa, 32.40921/-2.59711, 29-V-2008, S. Touil leg.: 5 exx. (ECWP); Tamlelt, Bouârfa, 32.42330/-2.58946, 1056 m, 6-V-2015, S. Touil leg.: 1 ex. (ECWP); Road Bouàrfa-Figuig, 32.25457/-1.71231, 1275 m, 24-V-2015, A. François, M. García-París & J.L. Ruiz leg.: 4 exx. (ECWP & JLR); Mengoub, Road Bouârfa-Bouanane, 32.2695/-2.35015, 1002 m, 26-V-2015, A. François, M. García-París & J.L. Ruiz leg.: 12 exx. (ECWP & JLR). ***Croscherichia mozabita*** (Pic, 1897): MOROCCO: Figuig, 10-V-1997, G. Chavanon leg: 3 exx. (JLR); Anoual, 32.64221/-3.12263, 1365 m, 24-V-2008, A. François leg.: 7 exx. (ECWP); Sud Matarka, 32.62186/-2.85240, 1193 m, 24-V-2008, A. François leg.: 1 ex. (ECWP); Bouârfa, 32.48182/-2.72814, 5-VI-2008, S. Touil leg.: 5 exx. (ECWP); Bouârfa, 32.48212/-2.7282, 12-VI-2008, S. Touil leg.: 6 exx. (ECWP); Bouârfa, 32.40936/-2.59689, 29-V-2008, S. Touil leg.: 2 exx. (ECWP); Bouârfa, 32.26789/-2.19231, 25-VI-2008, S. Touil leg.: 2 exx. (ECWP); Entre Outat el Haj et Matarka, 33.51521/-3.47590, 886 m, 13-VI-2010, A. François leg.: 4 exx. (ECWP); Road Bouàrfa-Figuig, 32.47915/-1.74761, 1170 m, 24-V-2015, A. François, M. García-París & J.L. Ruiz leg.: 14 exx. (ECWP & JLR); Mengoub, Road Bouârfa-Bouanane, 32.2695/-2.35015, 1002 m, 26-V-2015, A. François, M. García-París & J.L. Ruiz leg.: 13 exx. (ECWP & JLR). ***Croscherichia paykulli*** (Billberg, 1813): MOROCCO: Zaio, prov. Nador, V-1975, C. Peláez leg.: 2 exx. (JLR); Tleta de Oued Laou, prov. Tetouan, 9-VI-1991, J.M. Ávila leg.: 19 exx. (JLR); Dar Driouch, Oued Kert, prov. Nador, 12-VI-1991, J.M. Ávila leg.: 2 exx. (JLR); Crrtra. Midelt-Rich, Col du Tarhemt, 1900 m, 26-VI-1992, J.M. Vela leg. 1 ex. (JLR); Ifrane, Medio Atlas, 29-VI-1992, J.M. Ávila leg.: 2 exx. (JLR); Castillo Karia-Arkemane, prov. Nador, 29-VI-1996, J.M. Guzmán leg.: 11 exx. (JLR); Larache, cercanías de Lixus, 10-V-1997, J.L. Ruiz leg.: 4 exx. (JLR); Guefoüt, 26-V-1998, G. Chavanon leg.: 1 ex. (JLR); Oujda, Route de Toussit, 15-VI-2000, G. Chavanon leg.: 13 exx. (JLR); Missour Al Baten, 33.23047/-3.87382, 13-VI-2002, J. Yvernault leg.: 1ex. (ECWP); La Mamora, Kenitra, 23-V-2004, J.L. Ruiz leg.: 13 exx. (JLR); Missour ECWP, 33.00722/-4.09760, 954 m, 21/05/2010, A. François leg.: 3 exx. (ECWP); Massa, Souss-Massa National Park, 13-IV-2009, F.J. Martínez leg.: 5 exx. (JLR); Missour ECWP, 33.00722/-4.09760, 954 m, 17/06/2009, H. Hdidou leg.: 1 ex. (ECWP); Marismas del Oued Lucus, Larache, 4 m, 23-V-2009, J.L. Ruiz leg.: 7 exx. (JLR); Sidi Bou-Ghaba, Mehdia, Kenitra, 5 m, 24-V-2009, 5 exx. (JLR) Missour ECWP, 33.00722/-4.09760, 954 m, 21/05/2010, A. François leg.: 3 exx. (ECWP); Entre Ain Bni Mattar et Maatarka, 34.03349/-2.16300, 967 m, 5-VII-2012, A. François & M. Sbai leg.: 1 ex. (ECWP); Maatarka, Oued Sidi Ali, 33.35562/-2.77507, 1225 m, 13-VI-2013, A. François & L. Castro leg.: 2 exx. (ECWP); Missour ECWP, 33.00722/-4.09760, 954 m, 12/05/2014, S. Boullenger leg.: 2 exx. (ECWP); Outat Ouled el Haj, 33.34978/-3.66961, 812 m, 21-V-2015, A. François, M. García-París & J.L. Ruiz leg.: 5 exx. (ECWP & JLR); Maatarka, road to Debdou, 33.71822/-3.03126, 1338 m, 22-V-2015, A. François, M. García-París & J.L. Ruiz leg.: 1 ex. (ECWP & JLR); Entre El Ateuf et Debdou, 33.85486/-3.03628, 1484 m, 22-V-2015, A. François, M. García-París & J.L. Ruiz leg.: 7 exx. (ECWP & JLR); Missour ECWP, 33.00467/-4.09993, 965 m, 23-VI-2015, A. François leg.: 2 exx. (ECWP). ***Croscherichia richteri*** Kaszab, 1957: IRAN: Belutschistan Jranshar Dünen Nordwest Rig Ispakeh 2-IV-1954 Richter ü Sehäuffele leg. // **Paratypus** female *Croscherichia richteri*, m., det. Dr Kaszab, 1956 // *Croscherichia richteri* Kaszab, Dr Kaszab det. 1957: 2 exx. (HNHM). ***Croscherichia salavatiani*** Kaszab, 1968: IRAN: 62 km 300 S Iranshar 14-IV-1965 // Museum Paris Mission Franco-Iranienne 1965 // **Paratypus** 1967 *Croscherichia salavatiani* Kaszab // *Croscherichia salavatiani* Kaszab, Dr Kaszab det. 1957: 1 ex. (HNHM); Iran Bandar Abbas 29-III-1965 25 à l’ouest près de la cote // Museum Paris Mission Franco-Iranienne 1965 // **Paratypus** 1967 *Croscherichia salavatiani* Kaszab // *Croscherichia salavatiani* Kaszab, Dr Kaszab det. 1957: 1 ex. (HNHM). ***Croscherichia sanguinolenta sanguinolenta*** (Olivier, 1811): EGYPT: Aegyptus Reitter (Coll. Reitter, *Croscherichia sanguinolenta* Ol., Dr Kaszab det. 1957): 1 ex. (HNHM). MOROCCO: Erfoud, Tafilalet, V-1998, Bouraada leg.: 2 exx. (JLR); Figuig, 10-V-1997, G. Chavanon leg.: 7 exx. (JLR); Figuig, Defilia, 11-V-1997, G. Chavanon leg.: 2 exx. (JLR); Figuig, 14-V-1999, G. Chavanon leg.: 2 exx. (JLR); Road Bouârfa-Tamlelt, 32.39393/-2.18189, 1055 m, 25-V-2015, A. François, M. García-París & J.L. Ruiz leg.: 10 exx. (ECWP & JLR). ***Croscherichia tigrinipennis*** (Latreille, 1827): EGYPT: Aegypte (Coll. Reitter, *Croscherichia tigrinipennis* Latr., Dr Kaszab det. 1957): 1 ex. (HNHM). MOROCCO: Merzouga, 15-V-1998, Bouraada leg.: 1 ex. (JLR); Road N-12 Alnif-Rissani, 31.230172/-4.736069, 815 m, 12/04/2017, J.M. Vela & G. Bastazo leg.: 5 exx. (JLR). ***Croscherichia vigintipunctata*** (Olivier, 1811): EGYPT: Le Caire, Hénon (Slg. R. Oberthür, Coll. E. Martin, Eing. Nr. 4 1956, *Croscherichia vigintipunctata* Ol., Dr Kaszab det. 1957): 4 exx. (HNHM). ***Croscherichia wartmanni*** (Pic, 1896): ALGERIA: Ain Sefra Coll. Reitter // **Paratypus** 1896 *Zonabris wartmanni* Pic. // *Croscherichia wartmanni* Pic, Dr Kaszab det. 1957: 1 ex. (HNHM); Aïn-Sefra (Slg. R. Oberthür, Coll. E. Martin, Eing. Nr. 4 1956): 1 ex. (HNHM). MOROCCO: Figuig, 16-V-1995, G. Chavanon leg: 1 ex. (JLR); Figuig, 10-V-1997, G. Chavanon leg: 4 exx. (JLR); Figuig, dunes à *Aristida*, 18-IV-1998, G. Chavanon leg: 3 exx. (JLR).

The morphological study was carried out on dry-mounted specimens using stereomicroscopy. Male specimens were rehydrated prior to the extraction of their genital structures, which were subsequently mounted on cardboard with dimethylhydantoin formaldehyde resin and pinned adjacent to their respective specimen. Measurements were taken using a micrometre coupled to one of the eyepieces, and camera lucida drawings were made of the structures. The ethanol-preserved paratypes were not measured to prevent possible tissue deterioration. Total specimen length was measured along the dorsal side from the anterior end of the labrum (with the head extended) to the elytral apex. Maximum width was measured as the width between the outer edges of the elytra at approximately three-fourths of the elytral length, also in dorsal vision. Photographs of live and recently dry-mounted specimens were taken with a digital camera. The terminology suggested by Pardo Alcaide (1948, 1950, 1954a, 1954c) and Bologna (1991) was used to describe the various parts of the male genitalia.

In the present study, the evolutionary species concept (as modified by Wiley 1978, 1981 and Wiley and Mayden 2000, see Ruiz and García-París 2015) was adopted to define species. Under this concept, a species can be defined as a unique lineage that maintains its identity with respect to other lineages and that presents its own evolutionary tendencies and historical fate. This concept combines the basic methodological implications of the phylogenetic species concept, in which lineages are defined objectively by the existence of reciprocal monophyly, with subjective aspects that allow for the characterization of the phyletic line, or species, as an independent unit. These aspects, evaluated in light of an informed hypothesis about the evolutionary future of the lineage, are critical for determining whether an independent lineage can be considered as a species (García-París et al. 2008).

For practical purposes and to separate groups of morphologically similar species of *Croscherichia*, the taxonomic scheme in the species key of Bologna and Coco (1991) was used. However, the resulting clusters may not correspond to monophyletic entities. The specific and subspecific composition of the genus proposed by Bologna and Coco (1991) and Bologna (2008) was also followed in the present study.

## Results

### Croscherichia armass

Taxon classificationAnimaliaColeopteraMeloidae

Ruiz, François & García-París sp. nov

F1B341F0-20E6-54CA-8F51-A74B947EC90A

http://zoobank.org/46F4A3E2-5A2D-451E-B059-FC39A8D9094E

**Figure 3.**
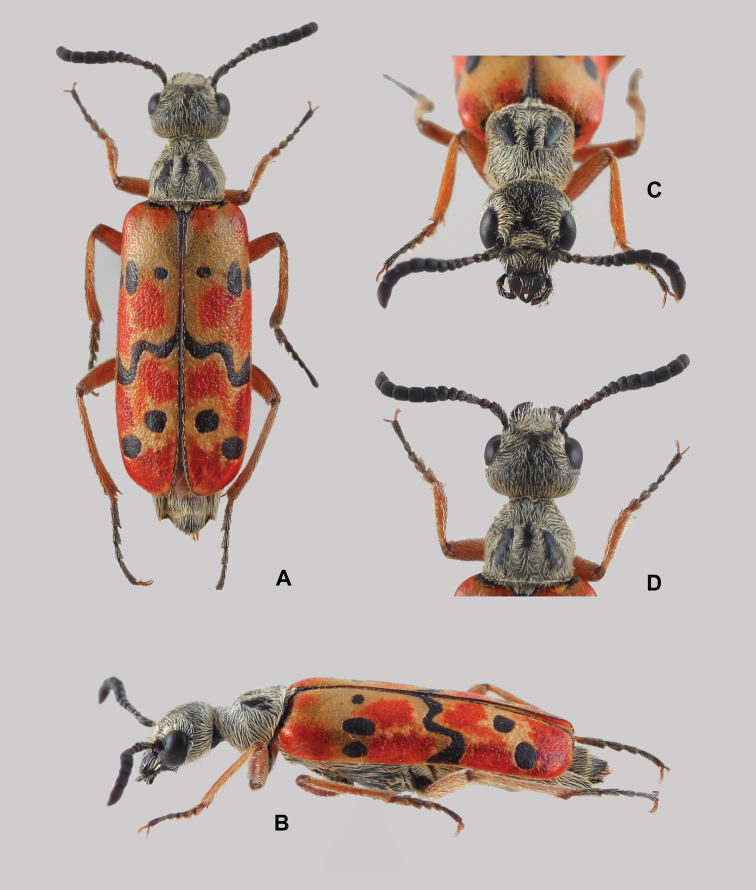
Holotype of *Croscherichia armass* sp. nov. **A** dorsal view **B** lateral view **C** frontal view **D** dorsal view of head and pronotum. Photographs by A. Sánchez-Vialas.

**Figure 4.**
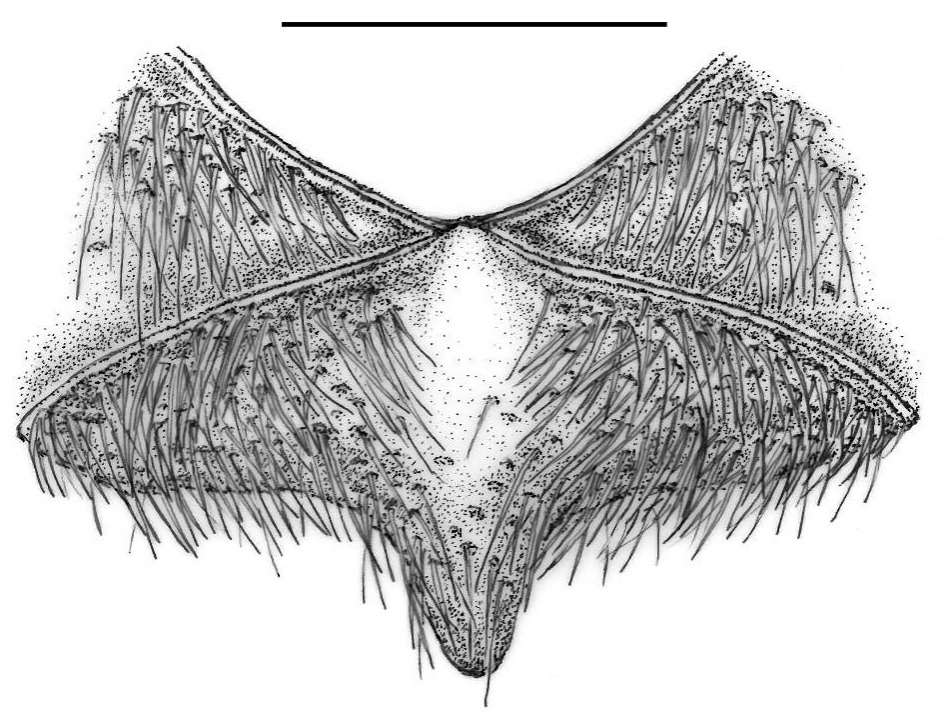
Mesosternum of *Croscherichia armass* sp. nov. Note the absence of a modified anterior medial area (*scutum* sensu Pardo Alcaide 1950). Scale bar: 0.5 mm. Drawing by JLR.

**Figure 5.**
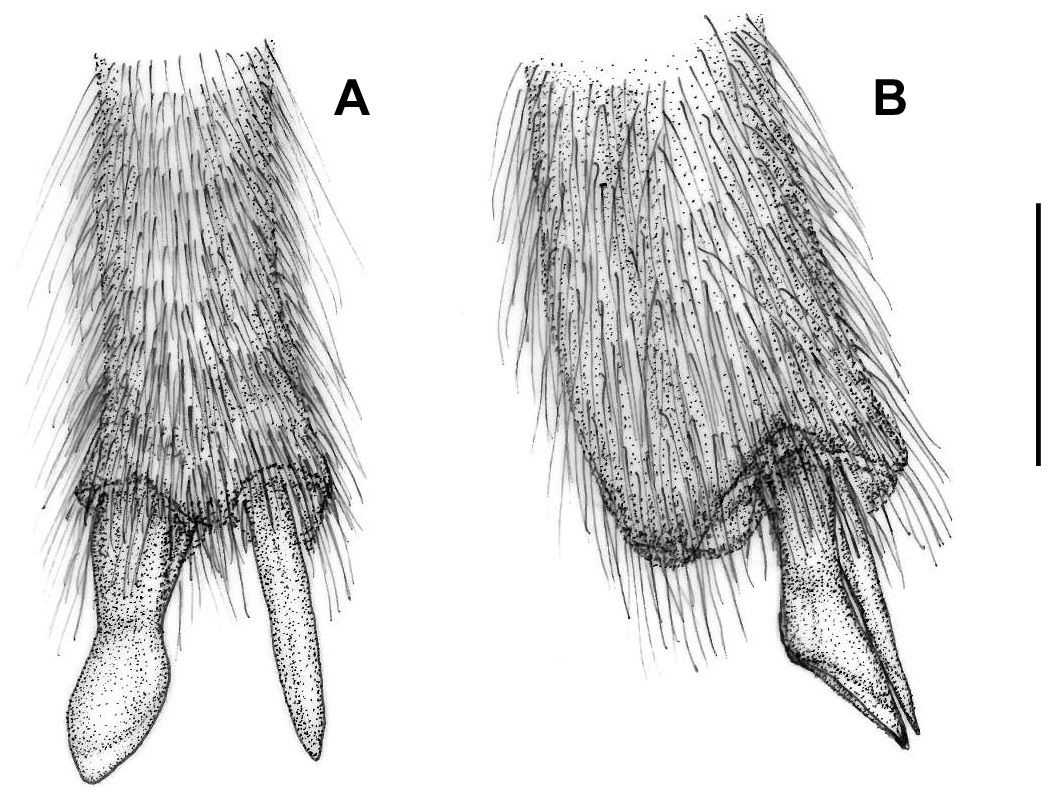
Metatibial spurs of *Croscherichia armass* sp. nov. **A** ventral view **B** lateral view. Scale bar: 0.5 mm. Drawing by JLR.

**Figure 6.**
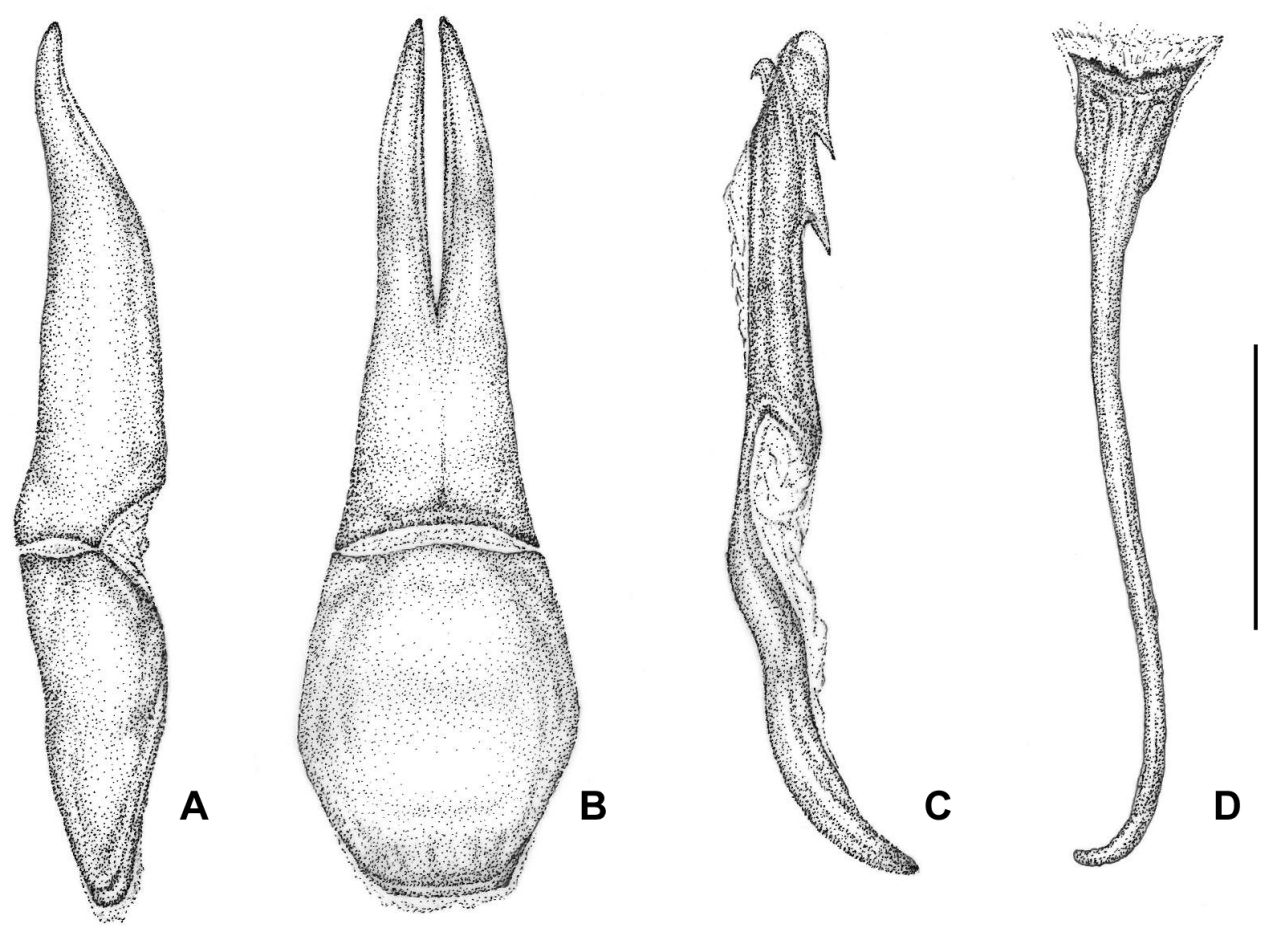
Aedeagus of *Croscherichia armass* sp. nov. **A** tegmen, lateral view **B** tegmen, dorsal view **C** median lobe, lateral view **D** spiculum gastrale. Note the narrow and elongate parameres and the middle lobe with two evident teeth in the ventral region that are subequal and clearly separated (type *isoharpagae* sensu Pardo Alcaide 1948). Scale bar: 0.5 mm. Drawing by JLR.

**Figure 7.**
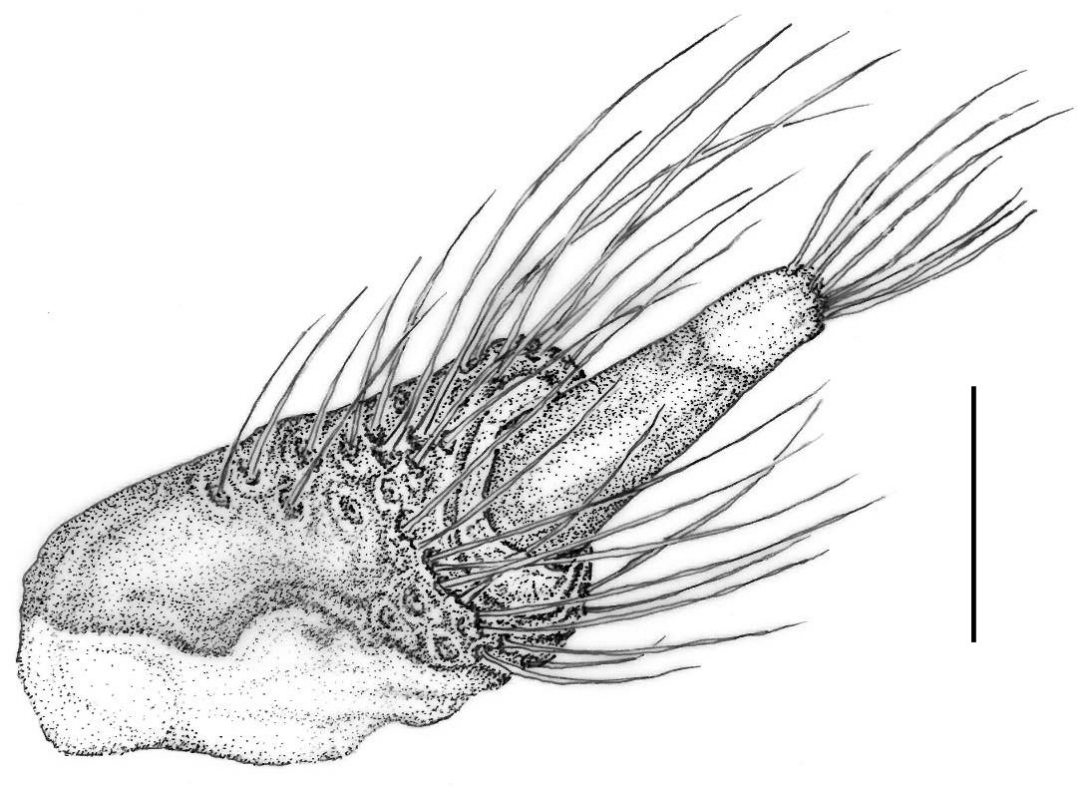
Valvifer and stylus of *Croscherichia armass* sp. nov. Scale bar: 0.5 mm. Drawing by JLR.

Figs 1A–D , 3A–D , 4 , 5A, B , 6A–D , 7

#### Type material.

***Holotype***: 1 male (dry-preserved) (Fig. 3), labelled: “10/09/2015, Missour ECWP, 33.00722/-4.0977600, A. François” / “954 m, Steppe à *Hammada scoparia*, Sur fleurs d’*Atriplex halimus*” (ivory labels, printed); “Holotypus, *Croscherichia armass* Ruiz, François et García-París des. 2018” (red label, printed). ***Paratypes***: 2 males, 4 females (dry-preserved), labelled: “12/09/2002, Missour Al Baten, 33.16433/-4.01064, J. Yvernault” / “032P1-2 BD019, Surface dépandage, *Salsola vermiculata*” (ivory labels, printed); 1 female, labelled: “13/09/2002, Missour Al Baten, 33.20696/-3.8697, J. Yvernault” / “Piège Barber 09p4-3, Surface d’epandage, *Salsola sieberi* et *Peganum hamala*, B032AB” (ivory labels, printed); 4 males, 11 females (2 males and 9 females dry-preserved, 2 males and 2 females preserved in ethanol), labelled: “10/09/2015, Missour ECWP, 33.00722/-4.0977600, A. François” / “954 m, Steppe à *Hammada scoparia*, Sur fleurs d’*Atriplex halimus*” (ivory labels, printed). All paratypes labelled: “Paratypus, *Croscherichia armass* Ruiz, François et García-París des. 2018” (red labels, printed).

#### Description of holotype (male):

***Total length***: 9.1 mm. Maximum width: 2.85 mm. General appearance elongated, stylized (Fig. 3). General coloration of the body tegument black, with orange legs, except tarsi that are chestnut brown, almost black. Antennae black, with antennomeres II, III, and the central area of I brownish. Elytra tricolored, reddish orange, with bands and black spots widely fringed with a halo of yellowish-ivory coloration lighter than the rest of the elytral integument; the contrast in coloration is especially noticeable *in vivo*. Very dense, greyish-silver body pubescence applied against the integument and mostly masking it, giving the body (except the elytra) a silvery grey appearance; pubescence on the elytra very short, with very fine and erect hairs.

***Head*** (Fig. 3B, C, D) widely rounded and broad in frontal view, without grooves or depressions, regular and weakly arched at vertex level, relatively narrow and short in lateral view; maximum width in front view: 1.75 mm; vertex to clypeo-frontal suture length: 1.26 mm; minimum width between the eyes (at the level of the front): 1.1 mm; short and broadly rounded temples, with a “maximum eye width (smaller diameter) to temple length” ratio of 1.02. Tegumentary surface glossy black with very thin microreticulation, presenting a small circular red spot that is diffuse but highly visible in the centre of the forehead at the level of the upper lobes of the eyes. Eyes convex but not very protuberant, slightly projecting from the natural convexity of the head, weakly notched at the level of the antennal insertions and with a very fine perimeter rim; larger diameter: 0.87 mm, smaller diameter: 0.51 mm. Forehead flat, except for a very small and diffuse central gibbosity at the level of the red spot, with a broad central zone, smooth and shiny, with a triangular outline, at the posterior end of which is the red spot. Vertex broadly arched, without a central longitudinal groove. Net, deep, arched clypeo-frontal suture. Clypeus transverse, 1.91 times wider than long, black, with the anterior half semimembranous and impunctate. Labrum relatively elongated, 1.4 times wider than long, with arcuate sides and the anterior margin slightly notched in the middle, with a narrow, weak, and diffuse central longitudinal line; black, except for an antero-central triangular zone with a semimembranous brownish-grey appearance, located just behind the notch. Cephalic capsule with fine but net and deep, dense punctures that are spaced apart by 0.5 to 1.0 times their diameter, except in the smooth disc region of the forehead; the highest density of punctures appears between the smooth zone and the inner margins of the eyes, as well as behind them and in a transverse band between the clypeo-frontal suture and the posterior margin of the antennal insertions; the lowest density occurs in the posterior region of the temples and vertex. Punctures of the clypeus restricted to the posterior half, puncture points slightly thicker and denser than those on the forehead, contiguous, almost masked by the dense setation; punctures of the labrum similar to that of the clypeus in a narrow basal region but thicker and scattered on the rest of the surface. Setation of the head greyish silver, relatively dense and semi-erect or stretched (according to zones), following the pattern of the punctures in which it is inserted; longer and denser setation around the eyes, side areas of the temples, and in a transverse strip located between the clypeo-frontal suture and the posterior margin of the antennal insertions; shorter and less dense setation on the sides and posterior regions of the frons and vertex; setation of the vertex and the posterior area of ​​the frons, stretched and forward-facing; setation of the sides of the forehead and the bands around the eyes semi-erect and directed towards the smooth disc; the setation inserted into the anterior transverse band of the forehead (between the antennal insertions and the clypeo-frontal suture), semi-erect and directed symmetrically toward the centre (of each half); setation of the temples mostly lying on the integument and directed forward, except for some long erect hairs located along the latero-ventral region that are directed downwards. Setation of the clypeus restricted to the posterior half, following the pattern of the punctures in which it is inserted, dense, semi-erected, and directed forward, with longer hairs on the sides and shorter hairs in the centre. Setation of the labrum very scarce and constituted by some hairs that are dispersed, fine and semi-erect, similar to those of the clypeal sides. Ventral region of the head, except the sides, smooth and hairless. Mandibles black, shiny, narrow, with their outer margins almost straight, except at the apical end where they are slightly curved; almost entirely hidden by the labrum in dorsal view. Maxillary palps short and black, with scarce setation similar to that of the labrum and subcylindrical segments slightly widened toward the extremity, the basal segment very short and the distal one truncated at the apex. Labial palps short and black with subconical palpomeres, the basal palpomere very small and the distal one truncated at the apex and slightly thickened towards the end. Dark brown ligule, notched at its anterior margin and with a deep longitudinal groove.

***Antennae*** (Fig. 3C, D) with 11 antennomeres: antennomere I black, with a dark brown coloration in the central zone; antennomeres II, III, and the basal zone of IV dark chestnut, bright; antennomeres IV (except in a basal ring) to X, black; antennomere XI black, with a dark brown area in the apex; antennomeres IV and V shiny, antennomeres VI to XI less shiny. Antennae relatively long, and when extended backwards, they reach the third quarter of the pronotum, weakly and gradually thickening toward the end from antennomere V. Length of the right antenna (extended): 2.92 mm; antennomere I elongated (length, l = 0.56 mm; maximum width, w = 0.19 mm; l/w: 2.94), slightly curved in the middle and slightly thickened at the end, with semi-lying, whitish, setation similar to that of the labrum but shorter; antennomere II short (l = 0.18 mm; w = 0.17 mm; l/w = 1.05), subcylindrical in the basal half and globose in the distal half, with setation similar to antennomere I but shorter; antennomere III narrow, elongated (l = 0.27 mm; w = 0.18 mm; l/w = 1.5), subconical, with pubescence similar to antennomere II; antennomeres IV (1 = 0.17 mm; w = 0.2 mm; l/w = 0.85) and V (1 = 0.18 mm; w = 0.24 mm; l/w = 0.75), short and wide, transverse, with setation similar to antennomere III but with scarcer and shorter hairs; antennomere VI transverse, wide (1 = 0.21 mm; w = 0.26 mm; l/w = 0.8), with very few hairs similar to those of antennomere V; antennomere VII subcylindrical, slightly wider at the distal end (1 = 0.24 mm; w = 0.26 mm; l/w = 0.92); antennomeres VIII (1 = 0.26 mm; w = 0.28 mm; l/w = 0.92), IX (1 = 0.28 mm; w = 0.26 mm; l/w = 1.07) and X (1 = 0.3 mm; w = 0.29 mm; l/w = 1.03) cylindrical and subequal; antennomere XI (1 = 0.5 mm; w = 0.27 mm; l/w = 1.85) cylindrical in the basal half and conical in the distal half, with a blunt tip; setation of antennomeres VI to XI hardly noticeable, whitish yellow, and applied to the tegument surface, with a few hairs longer, thin and erect.

***Pronotum*** (Fig. 3A, B, D) with glossy black tegument, with hardly noticeable fine microrreticulation; with a predominantly silver appearance due to the dense setation that covers the pronotal surface, except for a central depression and two smooth zones at its sides; almost as long as wide; length along the mid-line: 1.62 mm; maximum width, measured between the second and third pronotal fifth (at the level of the lateral angles): 1.65 mm; shape subquadrangular to trapezoidal in dorsal view, weakly truncated anteriorly; in lateral view, markedly sloping from lateral angles forward, backwards smoothly convex; lateral margins converging in the anterior-most two fifths and sinuated in the posterior-most three fifths; lateral angles well marked, protruding in dorsal view and rounded, like the anterior ones; posterior angles obtuse and rounded; basal region very weakly notched in the centre, directly facing the scutellum, with a very fine rim, masked by a line of dense, short, and semi-erect hairs directed towards the centre. Pronotal macrosculpture constituted by a central depression (located approximately in the pronotal third fifth) that appears as a longitudinal fossa with fuzzy borders, smooth, with a length of approximately 0.34 mm and a maximum width of 0.14 mm, and two broad slightly elevated symmetrical areas, smooth and hairless, ovoid in outline, widest in the posterior area, obliquely located on both sides of the discal depression, almost reaching the pronotal basis. Pronotal sculpture barely visible, mostly hidden by dense setation, consisting of thin, net punctures similar to those of the forehead, dense, subconfluent, uniformly distributed throughout the surface except for the central depression and the two smooth areas. Setation silvery, dense, relatively long and thick, mostly applied against the pronotal surface, and distributed according to the pattern of punctures in which it is inserted; setation directed backwards in the anterior-most two fifths, towards the centre on the sides, and forward in the posterior fifth; the setation surrounding the central depression is semi-erect and radially and outwardly directed; the setation located along the inner margins of the smooth areas is semi-eccentric and directed forward; the ends of the hairs from these two areas (central depression and smooth areas) overlap, giving rise to two bands of hairs with the appearance of a long tuft.

Scutellum hemi-elliptic, rounded along its posterior margin, with black and shiny integument, very densely and finely punctured, with a setation that completely covers the surface, similar to that of the pronotum, giving it a silvery appearance.

***Elytra*** (Fig. 3A, B) elongate, subparallel, with the humeral region slightly protruding and broadly rounded; length from the base of the pronotum to the apical end: 6.15 mm; maximum width, measuring both elytra together at the third quarter: 2.9 mm; semi-glossy surface; background coloration reddish orange, with a band and several black spots with well-defined contours surrounded by a broad, diffuse, yellowish-ivory halo; with the following design: (1) a small basal hemi-elliptic black spot contiguous to the humeral (prehumeral) region, which is almost completely covered by the posterior angular margins of the pronotum, that continues toward the centre as a thick line that borders the scutellum and, from the end of it, extends towards the elytral suture; (2) an anterior transverse series of three elliptic spots, clearly separated, aligned and located approximately in the posterior area of ​​the anterior third of the elytron; the inner spot almost rounded and well separated from the suture, with its major axis (Ma) positioned transversely (Ma = 0.32 mm; minor axis, ma = 0.24 mm, measured on the left elytron); the central spot, closer to the external spot, is the largest, with its Ma positioned longitudinally (Ma = 0.70 mm, ma = 0.40 mm); the external spot, similar to but smaller than the central one (Ma = 0.50 mm, ma = 0.36 mm), is located in the lateral declivity of the elytron, barely visible dorsally and well separated from the external edge of the elytron; (3) a narrow central zigzagging band (width in the middle = 0.30 mm, maximum width in the contact zone with the elytral edge = 0.68 mm) located slightly behind the centre of the elytron; this band joins the elytral suture and external edge; (4) two large posterior blotches, obliquely arranged, located in the posterior third of the elytron; the internal elliptical blotch (Ma = 0.62 mm, ma = 0.48 mm) is positioned anterior to the external, almost rounded one (Ma = 0.49 mm, ma = 0.46 mm) and well separated from the elytral edge; and (5) a thin black longitudinal line running through the entire suture and extending through the inner half of the elytral apex, its widest point is just below the scutellum in the area of attachment to the median band. Elytral punctures relatively coarse, subconfluent, dense and uniformly distributed over most of the elytral surface except in the periscutellar and humeral region where they are less dense and smaller. Setation of the elytral surface scarcely perceptible but deciduous and composed of very short yellowish-white hairs, directed vertically or subvertically; suture and elytral margin with a line of longer hairs (approximately two to three times the length of surface hairs), lying backwards.

***Mesopleurae*** without a careniform fold or rim along the anterior margin; setation dense and directed backwards, covering most of the mesopleural surface except along the central zones contiguous to the mesosternum, which are smooth and almost hairless. Mesosternum (Fig. 4) black, shiny and very finely microreticulated, without a modified anterior medial area (*scutum* or “central shield” sensu Pardo Alcaide 1950, 1954a); slightly elevated at the centre of the anterior edge, with the anterior margins rimmed, forming an open angle; lateral branches of the mesosternum relatively short and narrow; central posterior projection in blunt point between mesocoxae; discal and anterior areas of mesosternum smooth and hairless. Mesosternal punctures fine, scarce, and restricted to mainly the lateral branches and the posterior projection. Setation long, similar to that of the mesopleurae, lying backwards, following the pattern of the punctures in which they are inserted.

***Ventral region*** of the body with black integument, shiny, finely microreticulated, with fine and very dense punctures, subconfluent, hidden under the setation; setation very dense, evenly distributed and longer than that of the pronotum, giving the body a silver appearance except along the narrow mid-longitudinal band of the metasternum, which is smooth and hairless. Last abdominal ventrite with a deep and wide V-shaped central notch in its posterior margin, with a setation much less dense than the rest of the abdominal ventrites.

***Legs*** thin and narrow, with reddish-orange femora and tibiae, somewhat obscured distal ends, brownish-red trochanters that are slightly orange at the ends, black coxae that turn brownish red towards the apex; tarsi, dark brown almost black, except for the first protarsomere and the basal half of the first meso- and metatarsomeres, which are reddish brown; relatively short protarsi (length excluding claws = 1.39 mm), with protarsomere I short and wide, subconical in dorsal view (length, l = 0.30 mm; maximum width, w = 0.22 mm; l/w = 1.36), protarsomeres II (l = 0.22 mm; w = 0.16 mm; l/w = 1.37), III (l = 0.20 mm; w = 0.16 mm; l/w = 1.25) and IV (l = 0.18 mm; w = 0.12 mm; l/w = 1.5) subequal to protarsomere I, although gradually becoming smaller; protarsomere V (l = 0.5 mm; w = 0.14 mm; l/w = 3.57) subcylindrical, narrow and elongated; mesotarsi similar to but slightly more elongated (l = 1.58 mm) than protarsi and with slightly narrower tarsomeres; metatarsi longer and narrower than mesotarsi (l = 2.02 mm), with narrower tarsomeres. Leg punctures very dense and thin, slightly smaller than those on the ventral region of the body; setation whitish yellow, dense and thinner and shorter than that of the ventral region, almost lying on the surface, with the highest density on the tibiae and the inferior side of the femora; internal side of the anterior tibiae with a band of whitish hairs shorter and slightly denser than on the rest of the protibiae, hairs on the distal half of the outer carina slightly more erect and denser than the rest, uniform in size, without longer hairs. External apical end of the protibiae terminates in a small or narrow digitiform expansion almost covered entirely by setation. Tarsi with tarsomeres I to V bearing a small hirsute brush on the underside; on tarsomere V, the brush only occupies the proximal half; hairs on the protarsomeres are exclusively white, while those on the meso- and metatarsomeres are a mixture of white and dark brown. Apical spines of pro- and mesotibiae very small and narrow, subequal, with a blunt tip; apical internal spine of the metatibiae similar to that of the pro- and mesotibiae (length = 0.21 mm), the external one slightly longer and thicker (length = 0.22 mm), subcylindrical, obliquely truncated at the apex, weakly spatulate but slightly widened distally (Fig. 5). Brown to orange claws, curved along the apical two-thirds, with a weak lower basal tooth; upper and lower lobes of similar length, the lower lobe narrower.

***Aedeagus*** (Fig. 6A–C) with narrow and elongated parameres, 1.55 times longer than the phalobase, with a deep and narrow central longitudinal cleft in the apical half in dorsal view; parameral lobes slightly curved, having a digitiform aspect in lateral view and narrow, laminar and acuminate towards the apex in dorsal view. Phalobase slightly widened in dorsal view and narrow in lateral view. Middle lobe narrow, with rounded apex, obliquely truncated in its dorsal-apical region and curved in its proximal half, with two evident teeth in the ventral region, subequal and clearly separated (type *isoharpagae* sensu Pardo Alcaide 1948, 1950), near but well distanced from the apex. Dorsal-apical hook (*uncus* sensu Pardo Alcaide 1948, 1950), small, curved at the end, and with a sharp tip. Spiculum gastrale as in Figure 6D.

**Female**: Similar to the male but differing in the following features: protibiae with a small sharp tooth at the external apical end, with long and fine whitish semi-erect hairs along the outer edge, standing out from the short and lying setation of the surface; external side of the first four protarsomeres with long erect hairs, directed forward, similar to those on the outer edge of the protibiae, which also stand out from the short lying setation; last abdominal ventrite with a posterior margin that is not notched in the middle. Valvifer and stylus as in Figure 7.

#### Variability.

Not very marked but present in the following characters: total length, 7.3 to 10.1 mm, mean 8.6 mm (*N* = 19), males 7.6 to 9.1 mm, mean 8.2 mm (*N* = 5), females 7.3 to 10.1 mm, mean 8.7 mm (*N* = 14); maximum width, 2.2 to 3.7 mm, mean 2.8 mm (*N* = 19), males 2.2 to 2.8 mm, mean 2.6 mm (*N* = 5); females 2.3 to 3.7 mm, mean 2.9 mm (*N* = 14); frontal red spot with little variation in diameter and intensity; chromatic pattern of the antennae variable, particularly in the amplitude of the brown coloration: between antennomeres I and V, both inclusive: 26.3% (*N* = 19) (antennomere I presents a very dark basal region), between antennomeres II and V: 52.6% (sometimes the apical third of antennomere V is black), between II and IV: 5.3%, and between II and III: 15.8%, including the holotype; antennomeres VI–XI always black, although some specimens present a narrow basal brown ring on antennomeres VI and XI; punctures and setation of the head slightly variable in density, especially in the posterior area of the frons and in the vertex, with larger individuals showing greater density; smooth and glabrous pronotal areas that vary to a slight extent; elytral design generally constant, although there is a certain variation in the size of the spots of the anterior and posterior series (in the latter, the two rounded spots are joined in some specimens) and in the thickness of the sinuous median band, which, in some specimens, is interrupted in the middle; the external apical spine of metatibiae in larger specimens is visibly widened in the distal half. No variation is observed in the aedeagus of the studied males.

The coloration of live specimens varies markedly with that of the preserved specimens (Fig. 1C, D). Contrasts in the elytral coloration pattern are much more evident in live specimens, especially the ivory tones of the background and the reddish-orange coloration, which remains in only a few of the preserved specimens. In the specimens collected in 2002, the reddish coloration has been completely lost, and the elytra only retain a bicolored appearance with a uniform pale background and black spots and bands.

#### Etymology.

The word “armass” refers to the Tamazight (Bereber) name of the plant on which *C. armass* is usually found (*Atriplex halimus*).

#### Distribution and autoecological notes.

*Croscherichia armass* is only known from three nearby localities (separated by a maximum distance of about 35 km) in the region of Missour (Boulemane Province, Fès-Boulemane Administrative Region), east of the Eastern Middle Atlas in the valley or middle section of the Muluya River (Oued Moulouya) (Fig. 2): 1. Interior area of ECWP (Missour), 954 m, 33.01191N, 4.09693W. 2. Al Baten 1 (between Missour and Outat el Haj), 830 m, 33.20696N, 3.8697W. 3. Al Baten 2 (between Missour and Outat el Haj), 980 m, 33.16433N, 4.01064W.

The region is dominated by calcareous sedimentary materials: Quaternary alluvial plains with superficial soils. The area is within the mesomediterranean bioclimatic zone (Michalet 1991) and presents a lower arid ombroclime (mean annual rainfall: 159 mm in Missour, and 153 mm in Outat el Haj) and a marked continentality, particularly during cold winters (Le Houerou 1989). The habitat of the species’ area of occupation (*sensu*IUCN 2001) is, in general, steppes with little vegetation. The interior area of the ECWP-Missour) includes mostly open formations known as the Hammada steppes. Typical species occurring in this arid environment include *Hammada scoparia* (Pomel) Iljin, *Launaea arborescens* (Batt.) Murb., *Atriplex halimus* L., *Acanthorrhinum ramosissimum* (Coss. & Durieu) Rothm., and *Echinops spinosissimus* Turra, *Salsola* L. In addition, the species *Retama sphaerocarpa* (L.) Boiss., *Ziziphus lotus* (L.) Lam., and *Tamarix* L. (Quézel et al. 1994) are found along the wadi courses. The vegetation of the ECWP interior area is in an excellent state of conservation and presents a high level of diversity, mainly due to a perimeter enclosure that isolates it from anthropogenic activities, including livestock grazing. This space can, therefore, be considered as an authentic reserve for the region’s flora and fauna. The Al Baten area (both localities 1 and 2) (Fig. 1A) has lower vegetation cover and floristic diversity, and is in a poorer state of conservation, compared with the ECWP, a likely consequence of livestock (sheep and goats) grazing. This area is a steppe on an alluvial flood zone comprised of clay substrates (alluvial plain silt) that, depending on precipitation conditions, is used as a cereal field. Two species predominantly dominate the vegetation in Al Baten: *Atriplex halimus* L. and *Salsola gaetula* (Maire) Botsch. The conservation state of the Al Baten 2 vegetation (Fig. 1A) is better than that of Al Baten 1, particularly due to the presence of *Ziziphus lotus* and *Halogeton sativus* (L.) Moq. bushes.

Live *C. armass* specimens can be found in flight and actively feeding on flowers of the Chenopodiaceae*A. halimus* roughly between the hours of 10:00 and 16:00 (UTC). The coloration of *C. armass* blends in with the inflorescences of *A. halimus*, which can make locating specimens difficult. Bologna and Coco (1991) did not report *Croscherichia* feeding on any species of Chenopodiaceae. According to the scarce data available, the active period of *C. armass* adults is restricted to the end of summer, specifically to mid-September, which coincides with the blooming of *A. halimus*. Although the phenology of *C. armass* is not unique for *Croscherichia*, it is exceptional as only one other species, *C. delarouzei*, has been recorded as active in September (Bologna and Coco 1991). Moreover, to date, no other Mylabrini species have been reported as active in late summer in the Missour region. In fact, adults of Mylabrini in this region are only active during spring or, at most, until early summer, coinciding with the main plant flowering period (François, Ruiz and García-París, pers. obs. and in prep.).

## Discussion

*Croscherichia armass* has a very particular combination of morphological characters that distinguishes it from all other species of the genus. It has a relatively small size (mean = 8.3 mm, range: 7.3–10.1 mm); a black head, pronotum, and ventral region; reddish-orange legs with darkened tarsi; black antennae except for the proximal-most three to four antennomeres, which have a dark reddish-brown colour; and a dense and silvery body setation lying over most of the body integument. The broad head has a red spot on the frons and a dense and relatively thick setation on the integument. The outer margins of the mandibles are straight, pointed, and protrude from the labrum (at approximately one third of its length). The antennae are relatively short and black except for antennomeres II or III to V (rarely including antennomere I and the base of VI), which are dark or reddish brown. The pronotum is as broad as it is long and is neither elongated or narrowed at the anterior margin. It is characterized by a weak depression in its anterior third, a marked central longitudinal depression, and two well-defined smooth and glabrous areas lateral to the mid-line. The pronotal setation is silvery, thick, and dense against the integument, masking it for most part. The mesosternum has an angulate anterior margin and is covered with setation lying along its lateral branches. The external metatibial spur is short, subcylindrical, and not spatulate; it is also slightly widened at its distal end and truncated obliquely at the apex. The leg claws have upper and lower lobes of equal length that are markedly curved. The last abdominal sternum of the male has a deep cleft in its posterior margin. The elytral design of *C. armass* is unique within *Croscherichia*: tricolored, with a small prehumeral black spot, a narrow black zigzagging central band, and two rows of black rounded spots. The anterior row is comprised of three spots and the posterior one of two spots that neither touch the suture nor the external margin of the elytron.

The general appearance of *C. armass* resembles that of some North African species of *Ammabris*, particularly *Ammabris boghariensis* (Raffray, 1873) and, to a lesser extent, *A. avilai* (Ruiz & García-París, 2008). The three species are similar in the following features: elytral coloration, mandible morphology (straight and pointed outer margin), the presence of a red spot on the frons, pronotal sculpture (a central longitudinal groove with smooth areas lateral to it), and the overall silvery setation on the integument (see Ruiz and García-París 2008). However, the new species can be assigned to *Croscherichia*, and distinguished from *Ammabris*, by the following characters: the morphology of the external metatibial spur (subcylindrical, a little broadened distally, and truncated at the apex); the lack of a modified antero-central area in the mesosternum (“*mesosternal scutum*” *sensu*Pardo Alcaide 1948, 1950, 1954a, 1954c) that presents an angulate anterior margin; male genitalia with narrow parameres but without longitudinal groves along the parameral lobes; and the presence of two subequal, clearly separated teeth in the ventral region of the median lobe that are close to, but well distanced from, the apex.

According to the identification key of Bologna and Coco (1991), *Croscherichia* species with a black pronotum [the only species with a reddish-orange pronotum is *C. tigrinipennis* (Latreille, 1827)] can be divided into one of two groups based on antennal coloration. *Croscherichia armass* can be included in the group characterized by black or dark brown antennae [i.e., *C. delarouzei* (Reiche, 1866), *C. gilvipes* (Chevrolat, 1840), *C. goryi* (Marseul, 1870), *C. litigiosa* (Chevrolat, 1840), *C. mozabita* (Pic, 1897), *C. paykulli* (Billberg, 1813), *C. quadrizonata* (Fairmaire, 1875), *C. sanguinolenta* (Olivier, 1811), and *C. wartmanni* (Pic, 1896)] versus the one characterized by orange antennal antennomeres [i.e., *C. albilaena* (Bedel, 1899), *C. bedeli* (Bleuse, 1899), *C. femorata* (Klug, 1845), *C. fulgurita* (Reiche, 1866), *C. richteri* Kaszab, 1957, *C. salavatiani* Kaszab, 1968, and *C. vigintipunctata* (Olivier, 1811)].

*Croscherichia armass* can be further grouped with species having claws with upper and lower lobes of similar length, which includes *C. paykulli*, from the Maghreb (from Morocco to Libya); *C. delarouzei*, from Palestine, Israel, Jordan, and Lebanon; two subspecies of *C. sanguinolenta*, *C. s. sanguinolenta*, widely distributed throughout arid and semi-arid zones of North Africa, Senegal, and the Near East to Iran, and *C. s. arabica* Bologna & Coco, 1991, only known from Saudi Arabia; and *C. gilvipes*, distributed throughout arid regions of North Africa and the Near East (Israel, Jordan, and Syria) (Bologna and Coco 1991, Bologna 2008). *Croscherichia paykulli*, *C. delarouzei*, and the two subspecies of *C. sanguinolenta* can be easily distinguished from *C. armass* as these species have black or dark brown legs, black and hirsute setation (head, pronotum, and ventral region), distinct pronotal macrosculptures that lack smooth and glabrous lateral areas, clearly spatulate external metatibial spurs, and different elytral designs (see Bologna and Coco 1991). *Croscherichia armass* and *C. gilvipes* both have orange coloration on their legs, but the two species differ markedly in many other traits. Compared with *C. armass*, *C. gilvipes* has shorter antennae, jaws with curved external margins, and a whitish body setation that is thinner, slightly denser, and more lanuginose, and that does not conceal the tegument (Bologna and Coco 1991). In addition, the pronotal sculpture of *C. gilvipes* does not have smooth lateral areas, and the external metatibial spur is spatulate. Finally, its elytral setation is longer and denser, and the elytral chromatic design consists of distinctive internal spots that contact the suture (Bologna and Coco 1991).

*Croscherichia sonyae*, assigned with reservations to *Croscherichia* (Bologna and Pinto 2002), is endemic to Saudi Arabia and the United Arab Emirates (Bologna and Turco 2007, Bologna 2008, Batelka and Geishardt 2009). This species differs markedly from *C. armass*: it has orange antennae in which antennomeres V to VII are subtrapezoidal, making them stand out from the other antennomeres, protarsi with short and wide tarsomeres, overall scarce setation (head, pronotum, and ventral region), and a clearly banded elytral pattern (Kaszab 1983, Bologna and Pinto 2002, Bologna and Turco 2007, Batelka and Geishardt 2009).

Bologna and Coco (1991) based phylogenetic relationships within *Croscherichia* on an analysis of 26 morphological characters, which recovered 12 poorly supported groups. On the basis of morphology, *C. armass* most resembles the *C. delarouzei* and the *C. femorata*-*C. salavatiani* groups. *Croscherichia delarouzei* and *C. armass* share the derived condition of having elongated and pointed mandibles that extend beyond the labrum. *Croscherichia femorata*, *C. salavatiani* and *C. armass* share the derived state of having an unspatulated external metatibial spur. *Croscherichia armass* differs from *C. femorata*, only known from Saudi Arabia, and from *C. salavatiani*, widely distributed from the Arabian Peninsula to Pakistan (Bologna and Coco 2001, Bologna and Turco 2007, Bologna 2008), as these species present orange antennae, a whitish body setation that is much less dense, mandibles with curved outer margins, and a clearly banded elytral design (Bologna and Coco 1991, Batelka and Geishardt 2009).

Prior to the present study, nine *Croscherichia* species (i.e., *C. bedeli*, *C. fulgurita*, *C. gilvipes*, *C. litigiosa*, *C. mozabita*, *C. paykulli*, *C. sanguinolenta*, *C. tigrinipennis*, and *C. wartmanni*) were known for Morocco. None of these species are endemic to the country and, with the exception of *C. paykulli* (widely distributed throughout Morocco), all are restricted to arid or semi-arid zones in the south or the east (Pardo Alcaide 1954a, 1961, Kocher 1956, Bologna and Coco 1991, Ruiz and López-Colón 1996, Bologna 2008). With the discovery of *C. armass*, the only Moroccan endemic species of *Croscherichia* reported thus far, the number of species present in Morocco increases to 10, and the number of currently recognized species of *Croscherichia* increases to 19.

With the recent discoveries of new blister beetle species endemic to Morocco (Ruiz and García-París 2008, 2009, 2015, Černý and Bologna 2018, this work), the proportion of endemism has increased in the country. Given the large number of currently recognized species and the relatively high rate of endemism within the family, this trend is only likely to increase, making Morocco a hotspot for Meloidae diversity.

## Supplementary Material

XML Treatment for Croscherichia armass
